# Prevention of Preterm Birth by Cervical Pessary Combined with Vaginal Progesterone: a Systematic Review and Meta-analysis with Trial Sequential Analysis

**DOI:** 10.1007/s43032-022-00926-x

**Published:** 2022-03-29

**Authors:** Yanyan Zhuang, Huan Li, Quan Na, Shaowei Yin, Na Li

**Affiliations:** Department of Gynaecology and Obstetrics, Shengjing Hospital of China Medical University, No.36 Sanhao Street, Shenyang, 110004 China

**Keywords:** Preterm birth, Vaginal progesterone, Cervical pessary, Perinatal outcome, Delivery

## Abstract

This study was to assess the effectiveness of cervical pessary combined with vaginal progesterone for the prevention of preterm birth (PTB). Ten studies about singleton [five randomized controlled trials (RCTs), vs vaginal progesterone; four cohorts, vs vaginal progesterone; two cohorts, vs cervical cerclage + vaginal progesterone] and two cohort studies about multiple pregnancies (vs vaginal progesterone) were included after searching electronic databases. For singleton pregnancies, the meta-analysis of three non-RCTs [relative risk (RR) = 0.41, *p* = 0.001] or total trials in non-Asian country (RR = 0.56, *p* = 0.03) revealed that compared with vaginal progesterone alone, cervical pessary + vaginal progesterone treatment had significant effectiveness on preventing PTB < 34 weeks, but not for five RCTs; meta-analysis of two trials showed that cervical pessary + vaginal progesterone had no significant prevention effects of PTB compared with cervical cerclage + vaginal progesterone. For multiple pregnancies, meta-analysis of two trials showed that compared with vaginal progesterone, cervical pessary + vaginal progesterone treatment increased neonatal birth weight (standardized mean difference = 0.50, *p* = 0.01). Trial sequential analysis implied additional studies were required. Four studies vs other controls (pessary, three-combined, tocolysis, conservative or no treatment; one study, each) were selected for systematic review. In conclusion, cervical pessary combined with vaginal progesterone may be safe and effective to prevent PTB in singleton pregnancies and increase neonatal birth weight in the multiple pregnancies compared with vaginal progesterone alone.

**Supplementary Information:**

The online version contains supplementary material available at 10.1007/s43032-022-00926-x.

## Introduction

Preterm birth (PTB) is a common condition encountered in obstetrics, affecting 10.6% of pregnant women worldwide (equating to an estimated 14.84 million births) [1]. Compared with neonates born at term, preterm neonates are at increased risks of death and developing various complications [i.e., intraventricular hemorrhage (IVH), cerebral palsy, respiratory distress syndrome (RDS), necrotizing enteritis, sepsis, retinopathy of prematurity (ROP), chronic lung disease, neonatal infection and patent ductus arteriosis] [2, 3]. The supportive care for these premature infants generates considerable costs to families and healthcare systems [4]. Therefore, it is of great clinical importance to take effective and safe measures for the prevention of PTB.

Currently, three strategies are commonly recommended for the management of PTB in clinic, including cervical pessary, cervical cerclage, and progesterone. However, the effectiveness of a single intervention seems to be limited according to meta-analysis results: Xiong et al. integrated eight studies about singleton and six studies about twin pregnancies and concluded that the use of cervical pessary did not reduce the risk of PTB < 34 weeks, < 37 weeks, < 28 weeks of gestation and did not improve perinatal outcomes [5]. A pooled analysis of five studies with 419 asymptomatic singleton gestations done by Berghella et al. showed that cervical cerclage could not provide benefit in preventing preterm delivery or improving neonatal outcomes [6]. In the study of Jarde et al. which performed a meta-analysis of 23 studies, none of the interventions (progesterone, cerclage or pessary) were reported to have significant prevention effects on PTB at < 34, < 37 weeks of gestation and neonatal death for women with twin pregnancies compared to the control group [7]. Therefore, some scholars suggested to using a combined therapy [8–12]. Although there were systematic review and meta-analysis studies to assess the efficacy of a combined therapy for PTB prevention [13, 14], the evidence available was extremely insufficient (only three studies published before 2018 were included in these two studies, respectively). Furthermore, unlike cervical pessary and progesterone, cervical cerclage is a surgical procedure that is invasive, high-cost [15], and causes adverse complications (i.e., bleeding, infection) [16]. Thus, newly published studies about a combined therapy during 2018–2021 years mainly attempted to confirm the effects of cervical pessary plus progesterone on pregnancy and neonatal outcomes [17–27].

The primary objective of our study was to perform updated systematic review and meta-analysis to re-evaluate the effectiveness of cervical pessary combined with vaginal progesterone in the prevention of PTB and perinatal outcomes in women with singleton and multiple pregnancies. Furthermore, a trial sequential analysis (TSA) [28] was included to evaluate whether the statistical power of the currently available evidence was sufficient and the potential need for further studies.

## Materials and Methods

### Search Strategy

This meta-analysis was conducted by following the Preferred Reporting Items for Systematic Review and Meta-analysis (PRISMA) guidelines. Informed consent and ethical approval were not mandatory since all data available were based on previously published articles. Relevant studies were obtained through searching the electronic databases (including PubMed, EMBASE and Cochrane Library) from inception of each database to November 1, 2021. The search terms and their combinations were (“pessary”) AND (“progesterone” OR “combined” OR “plus”) AND (“preterm birth” OR “preterm labor”). No language restrictions were imposed. Additionally, the references of previously published systematic reviews, meta-analyses and our identified studies were manually checked to find other potentially eligible studies.

### Selection Criteria

Studies were included based on the participants, interventions, comparisons, outcomes, and study design criteria: (1) participants—study objects were pregnant women with a potential risk for PTB (such as a short cervix in the mid-trimester, history of PTB, spontaneous miscarriage, pregnancy after in vitro fertilization, multiple pregnancy, uterine anomalies or cervical surgery) (see the inclusion criteria of patients in each article; Table S1); (2) intervention and comparison—each study contained two groups. One underwent cervical pessary + progesterone combined treatment, while the other group received control intervention (progesterone, pessary, cerclage, three-combined, conservative or no treatment). The pessary types were not restricted; (3) outcomes—at least one pregnancy or neonatal outcome was reported; and (4) study design—randomized controlled trials (RCTs) or non-randomized cohort studies. Studies were excluded if they (1) were duplicate publications; (2) were case reports, reviews, protocols, letters and comments; (3) did not design a control group; and (4) did not report clinical outcomes.

### Data Extraction

Two authors independently collected the following data from the included studies: the first author’s name, year of publication, country, study design, sample size, intervention method, pregnancy, and neonatal outcomes. The pregnancy outcomes included gestational age (GA) at delivery, the incidence of PTB < 28 weeks, 32 weeks, 34 weeks, and 37 weeks of gestation and vaginal discharge. Neonatal outcomes included mean birth weight (in grams), incidence of low birth weight (LBW, < 2500 g), antenatal death, neonatal death, intrapartum death, perinatal death (defined as death at any period), neonatal intensive care unit (NICU) admission, RDS, IVH, ROP, sepsis, and composite adverse neonatal outcomes (defined as at least one of the following: necrotizing enterocolitis, IVH, RDS, bronchopulmonary dysplasia, ROP, sepsis and neonatal death). Disagreements were resolved by discussion until a consensus was reached.

### Quality Assessment

Two reviewers independently assessed the quality of each included study. Any disagreements were resolved through consensus. The risk of bias in RCTs was determined based on the Cochrane Handbook method [29] where seven domains were assessed (including random sequence generation, allocation concealment, blinding of participants and personnel, blinding of outcome assessment, adequate assessment of incomplete outcome, selective reporting avoided, and no other bias). For each study, each domain was categorized into “high,” “low,” or “unclear” risk. The quality of cohort studies was tested using the Newcastle–Ottawa Scale (NOS) [30] that included three aspects: selection (0–4 points), comparability (0–2 points), and exposure (0–3 points). A maximum of nine stars was given for each study: more than seven stars indicated high quality.

### Statistical Analysis

Statistical meta-analyses were performed by using the STATA statistical Packages (v13.0; STATA Corporation, College Station, TX, USA) and Review Manager software (v5.3; Cochrane Collaboration, Oxford, UK). Pooled relative risk (RR) and 95% confidence interval (CI) were calculated for dichotomous variables, while standardized mean difference (SMD) and 95% CI were for continuous outcomes. RR > 1 (or SMD > 0) indicated an increased risk fold in outcomes of the intervention group compared with the control group. The influence of the combined treatment on outcomes was thought to be statistically significant if the 95% CI did not overlap 1 and *p* value determined by *Z*-test < 0.05. Statistical heterogeneity across included studies was assessed using *I*-squared statistic (I^2^) and Cochrane’s *Q*-test. Combined effect size was calculated using a fixed-effects model when heterogeneity was not significant (*p* > 0.1 and *I*^2^ < 50%); otherwise, a random-effects model was applied (*p* < 0.1 and *I*^2^ > 50%). To explore the source of heterogeneity, subgroup analyses were carried out for variables with the number of studies larger than three based on the following factors: design (multiple-center or single-center), cervical length (> 25 mm or ≤ 25 mm), and ethnicity (Asian or non-Asian). Sensitivity analyses were implemented to reflect the influence of individual study on the overall estimate by removing each study in turn. Publication bias was evaluated by Egger’s linear regression test, with *p* < 0.05 defined as the statistical threshold.

Meta-analyses are prone to have type I errors (false-positive result) or type II errors (false-positive result) due to random errors (e.g., sparse data, repeated significance testing, and lack of power). Thus, TSA was also performed using TSA software package (v.0.9.5.5 beta; Copenhagen Trial Unit, Centre for Clinical Intervention Research, Copenhagen, Denmark) [28] to adjust the random error and estimate the required information size (RIS, the sample size required to detect or reject effects in meta-analyses). The RIS was calculated for the outcomes with the random-effects model based on type I error of 5% and power of 80%. The trial sequential monitoring boundaries (TSMBs) were constructed based on O’Brien-Fleming alpha-spending function. If the cumulative *Z*-curve entered the futility area, crosses TSMB, or reached RIS, it indicated the present level of evidence was firm and no additional studies were required; otherwise, the evidence was rated as absent and further studies were necessary.

## Results

### Study Selection

**Fig. 1:**
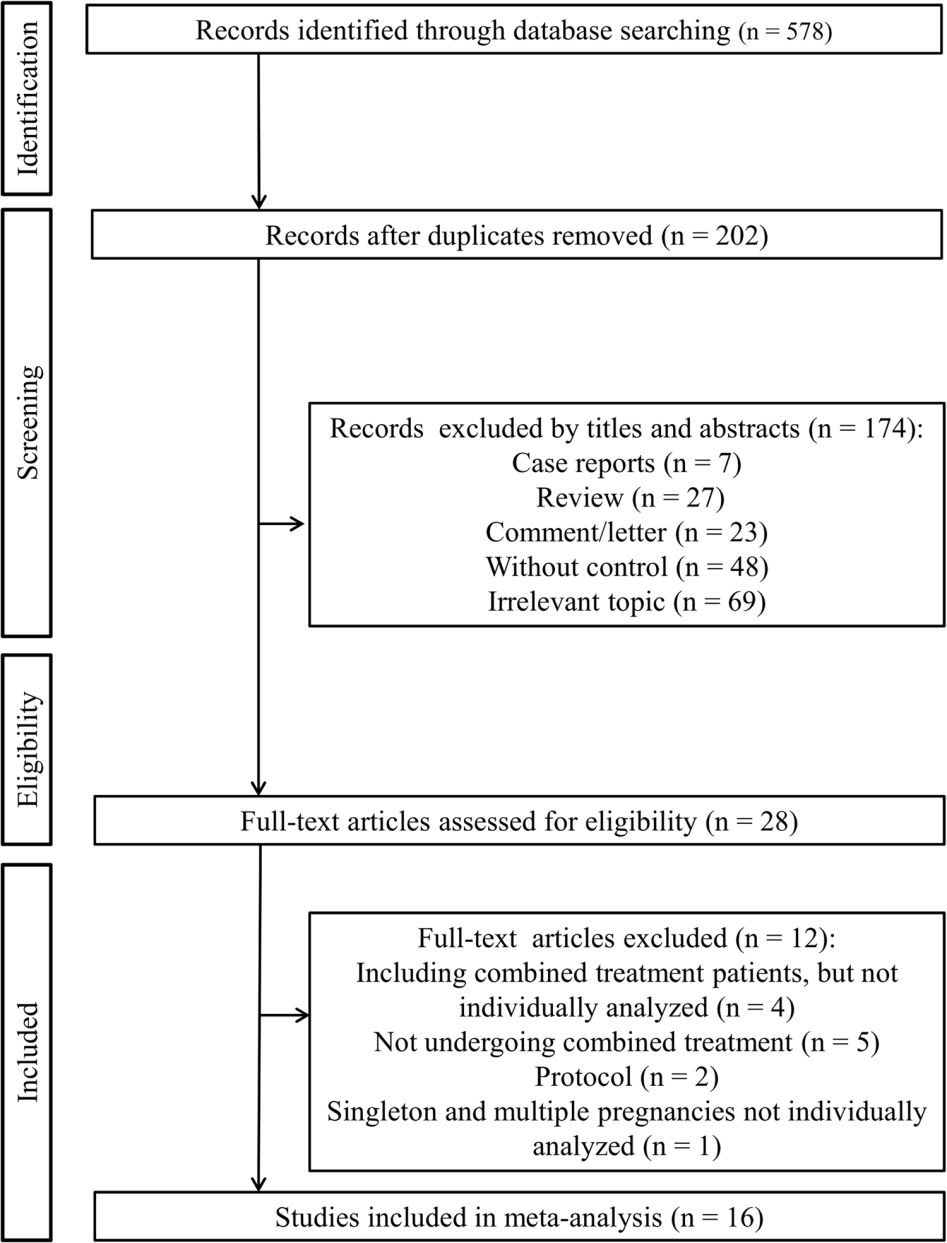
PRISMA flow diagram showing literature search process

The PRISMA flow diagram summarizes the selection procedure (Fig. 1). The literature searching in the electronic databases initially yielded 578 articles, of which 376 were duplicates. After reading the titles and abstracts, 174 references were excluded since they were case reports (*n* = 7), review (*n* = 27), comment/letter (*n* = 23), without control (*n* = 48), and irrelevant topics (*n* = 69). The remaining 28 studies were retrieved for full-text review, of which 12 papers did not meet the inclusion criteria for the following reasons: although cervical pessary combined with progesterone treatment was applied in partial patients, these patients were not individually analyzed in four studies; only cervical pessary or progesterone alone treatment, not combined treatment was used in five studies; two studies were protocols; singleton and multiple pregnancies were not individually analyzed in one study. Sixteen studies, with 1517 participants in the cervical pessary and vaginal progesterone combined therapy group and 1600 women in the control group were finally included [8–12, 17–27].

### Study Characteristics and Bias Assessment

Baseline characteristics of included studies are shown in Table 1. These included studies were published from 2015 to 2021. Four studies were conducted in Israel, three in Russia; two in Brazil; two in Italy; one in Japan, Iran, Germany, and the USA; and multiple centers of England, Slovenia, Portugal, Chile, Australia, Italy, Albania, Germany, and Belgium, respectively. The cervical length of patients was less than 25 mm in most of studies (15/16, 93.9%). Of 16 studies, 12 studies [8, 10–12, 18–22, 25–27] involved women with a singleton pregnancy and the other four [9, 17, 24, 27] included women with multiple pregnancies. Five RCT [10–12, 19, 25] and four non-RCT [18, 21, 26, 27] singleton studies included a control group receiving treatment with vaginal progesterone alone; two non-RCT singleton studies compared with a group receiving the combination of cervical cerclage and vaginal progesterone [18, 20]; only one non-RCT singleton study used the group receiving vaginal progesterone + cervical cerclage + cervical pessary [18], tocolysis (ritodrine hydrochloride and magnesium sulfate) [22] or cervical pessary alone [8] as the control, respectively. Two non-RCT [9, 23] multiple pregnancy studies included a control group receiving vaginal progesterone alone treatment; only one non-RCT multiple pregnancy used the group receiving conservative treatment [24] or no treatment [17] as the control, respectively. Thus, ten studies with patients in singleton gestations [10–12, 18–21, 25–27] and two studies [9, 23] with patients in multiple gestations were used for meta-analysis. The RR (or SMD) with 95% CI was calculated for other studies that could not be synthesized with others due to different controls [8, 17, 22, 24] to serve as a literature systematic review.

**Table 1:** Characteristics of included studies

	Study	Year	Country	Study design	Study group		Control group		Pregnancy	Cervical length	Outcomes
					No. of patients	Therapy	No. of patients	Therapy			
Meta	Mastantuoni E [25]	2021	Italy	RCT, single-center	32	Pessary + vaginal progesterone	29	Vaginal progesterone (capsules, 200 mg/day)	Singleton	≤ 25 mm	GA at delivery, PTB (< 28 weeks, < 32 weeks, < 34 weeks, < 37 weeks), birth weight, IVH, RDS, ROP, sepsis, composite outcome
	Barinov SV [19]	2020	Russia	RCT, single-center	81	Arabin cervical pessary + vaginal progesterone	136	Vaginal progesterone (capsules, 200 mg/day)	Singleton	32–36 mm	PTB (< 34 weeks, < 37 weeks)
	Saccone G [11]	2017	Italy	RCT, single-center	133	Arabin cervical pessary + vaginal progesterone	125	Vaginal progesterone (capsules, 200 mg/day)	Singletons	≤ 25 mm	PTB (< 34 weeks)
	Karbasian N [12]	2016	Iran	RCT, single-center	71	Arabin cervical pessary + vaginal progesterone	73	Vaginal progesterone (capsules, 400 mg/day)	Singleton	≤ 25 mm	GA at delivery, PTB (< 34 weeks, < 37 weeks), LBW, birth weight, antenatal death, neonatal death, perinatal death, NICU admission, composite outcome
	Nicolaides KH [10]	2016	England, Slovenia, Portugal, Chile, Australia, Italy, Albania, Germany, Belgium	RCT, multiple-center	465	Arabin cervical pessary + vaginal progesterone	467	Vaginal progesterone (capsules, 200 mg/day)	Singletons	≤ 25 mm	GA at delivery, PTB (< 28 weeks, < 32 weeks, < 34 weeks), LBW, antenatal death, neonatal death, perinatal death, NICU admission, IVH, RDS, ROP, sepsis, composite outcome
	França MS [26]	2021	Brazil	Cohort, single-center	95	AM cervical pessary + vaginal progesterone	33	Vaginal progesterone (capsules, 200 mg/day)	Singleton	< 25 mm	PTB (< 34 weeks), perinatal death, vaginal discharge
	Firichenko SV [27]	2021	Russia	Cohort, single-center	23	Arabin cervical pessary + vaginal progesterone	70	Vaginal progesterone (capsules, 200 mg/day)	Singleton	< 25 mm	PTB (< 37 weeks)
	Shor S[18]	2019	Israel	Cohort, multiple-center	120	Arabin cervical pessary + vaginal progesterone	110	Vaginal progesterone (capsules, 200 mg/day)	Singleton	≤ 25 mm	GA at delivery, PTB (< 37 weeks), birth weight
	Melcer Y[21]	2020	Israel	Cohort, multiple-center	94	Arabin cervical pessary + vaginal progesterone	108	Vaginal progesterone (capsules, 200 mg/day)	Singleton	≤ 25 mm	PTB (< 28 weeks, < 34 weeks), birth weight, vaginal discharge
	Shor S[18]	2019	Israel	Cohort, multiple-center	120	Arabin cervical pessary + vaginal progesterone	38	Cervical cerclage + vaginal progesterone (capsules, 200 mg/day)	Singleton	≤ 25 mm	PTB (< 37 weeks), birth weight
	Barinov SV[20]	2021	Russia	Cohort, multiple-center	161	Arabin cervical pessary + vaginal progesterone	79	Cervical cerclage + vaginal progesterone (capsules, 200 mg/day)	Singleton	≤ 25 mm	PTB (< 37 weeks), birth weight
	Fox NS [9]	2016	USA	Cohort, single-center	21	Arabin cervical pessary + vaginal progesterone	63	Vaginal progesterone (capsules, 200 mg/d)	Twin	< 25 mm	GA at delivery, PTB (< 34 weeks), birth weight, perinatal death, composite outcome
	Yaniv-Nachmani H [23]	2021	Israel	Cohort, multiple-center	68	Arabin cervical pessary + vaginal progesterone	78	Vaginal progesterone (capsules, 200 mg/d)	Twin	< 25 mm	PTB (< 34 weeks)
Systematic review	Shor S [18]	2019	Israel	Cohort, multiple-center	120	Arabin cervical pessary + vaginal progesterone	18	Vaginal progesterone + cervical cerclage + Arabin cervical pessary	Singleton	≤ 25 mm	GA at delivery, PTB (< 37 weeks), birth weight
	Tajima M[22]	2020	Japan	Cohort, single-center	43	Arabin cervical pessary + vaginal progesterone	53	Tocolysis (Ritodrine hydrochloride and magnesium sulfate)	Singleton	≤ 25 mm	GA at delivery, PTB (< 32 weeks, < 34 weeks, < 37 weeks), birth weight, RDS, composite outcome, vaginal discharge
	Stricker N [8]	2015	Germany	Cohort, single-center	53	Arabin cervical pessary + vaginal progesterone	53	Arabin cervical pessary	Singleton	≤ 25 mm	PTB (< 28 weeks, < 32 weeks, < 34 weeks, < 37 weeks), birth weight, NICU admission
	Zimerman A [24]	2018	Israel	Cohort, multiple-center	32	Arabin cervical pessary + vaginal progesterone	26	Conservative	Twin	< 25 mm	GA at delivery, PTB (< 28 weeks)
	França MS [17]	2020	Brazil	Cohort, single-center	25	AM cervical pessary + vaginal progesterone	32	No treatment	Twin	< 25 mm	GA at delivery, PTB (< 28 weeks, < 32 weeks, < 34 weeks, < 37 weeks), birth weight

*RCT* randomized controlled trials, *GA* gestational age, *PTB* preterm birth, *NICU* neonatal intensive care unit, *LBW* low birth weight, *IVH* intraventricular hemorrhage, *RDS* respiratory distress syndrome, *ROP* retinopathy of prematurity

The risk of bias of each study is shown in Table 2. Five domains in RCT studies had a low risk of bias. Except the study of Saccone et al. [11], most of studies were not blinded and thus, a high risk of bias was also present in domains of blinding of participants and personnel, and blinding of outcome assessment. The NOS score of cohort studies was seven for six articles and eight for five studies. These findings indicated the included literatures were overall of high-quality.

**Table 2:** Bias evaluation

RCTs^*^								
Study	Random sequence generation	Allocation concealment	Blinding of participants and personnel	Blinding of outcome assessment	Adequate assessment of incomplete outcome	Selective reporting avoided	No other bias	
BariHighv SV (2020)	Low	Low	High	High	Low	Low	Low	
Karbasian N (2016)	Low	Low	High	High	Low	Low	Low	
Nicolaides KH (2016)	Low	Low	High	High	Low	Low	Low	
Saccone G (2017)	Low	Low	Low	Low	Low	Low	Low	
Mastantuoni E (2021)	Low	Low	High	High	Low	Low	Low	
Non-RCTs^†^								
Study	Selection (score)				Comparability	Exposure (score)		
	Representativeness of the exposed cohort	Selection of the non-exposed cohort	Ascertainment of exposure	Demonstration that outcome of interest was not present at start of study	Comparability of cohorts on the basis of the design or analysis	Assessment of outcome	Follow-up long enough for outcomes to occur	Adequacy of follow-up of cohorts
França MS (2020)	☆	☆	☆	☆		☆	☆	☆
Shor S (2019)	☆	☆	☆	☆		☆	☆	☆
BariHighv SV (2019)	☆	☆	☆	☆	☆	☆	☆	☆
Melcer Y (2020)	☆	☆	☆	☆		☆	☆	☆
Tajima M (2020)	☆	☆	☆	☆	☆	☆	☆	☆
Stricker N (2015)	☆	☆	☆	☆	☆	☆	☆	☆
Fox NS (2016)	☆	☆	☆	☆	☆	☆	☆	☆
Yaniv-Nachmani H (2021)	☆	☆	☆	☆		☆	☆	☆
Zimerman A (2018)	☆	☆	☆	☆		☆	☆	☆
Firichenko SV (2021)	☆	☆	☆	☆	☆	☆	☆	☆
França MS (2021)	☆	☆	☆	☆		☆	☆	☆

^*^The Cochrane risk-of-bias tool used for RCTs

^†^The Newcastle Ottawa Scale used for non-RCTs

☆One star was awarded if the study met the criteria of each item in the Newcastle Ottawa Scale. The total number of star in all items represented the high quality of each study

### Analysis for Outcomes in Women with a Singleton Pregnancy

#### Cervical Pessary + Vaginal Progesterone vs Vaginal Progesterone: RCTs

**Fig. 2:**
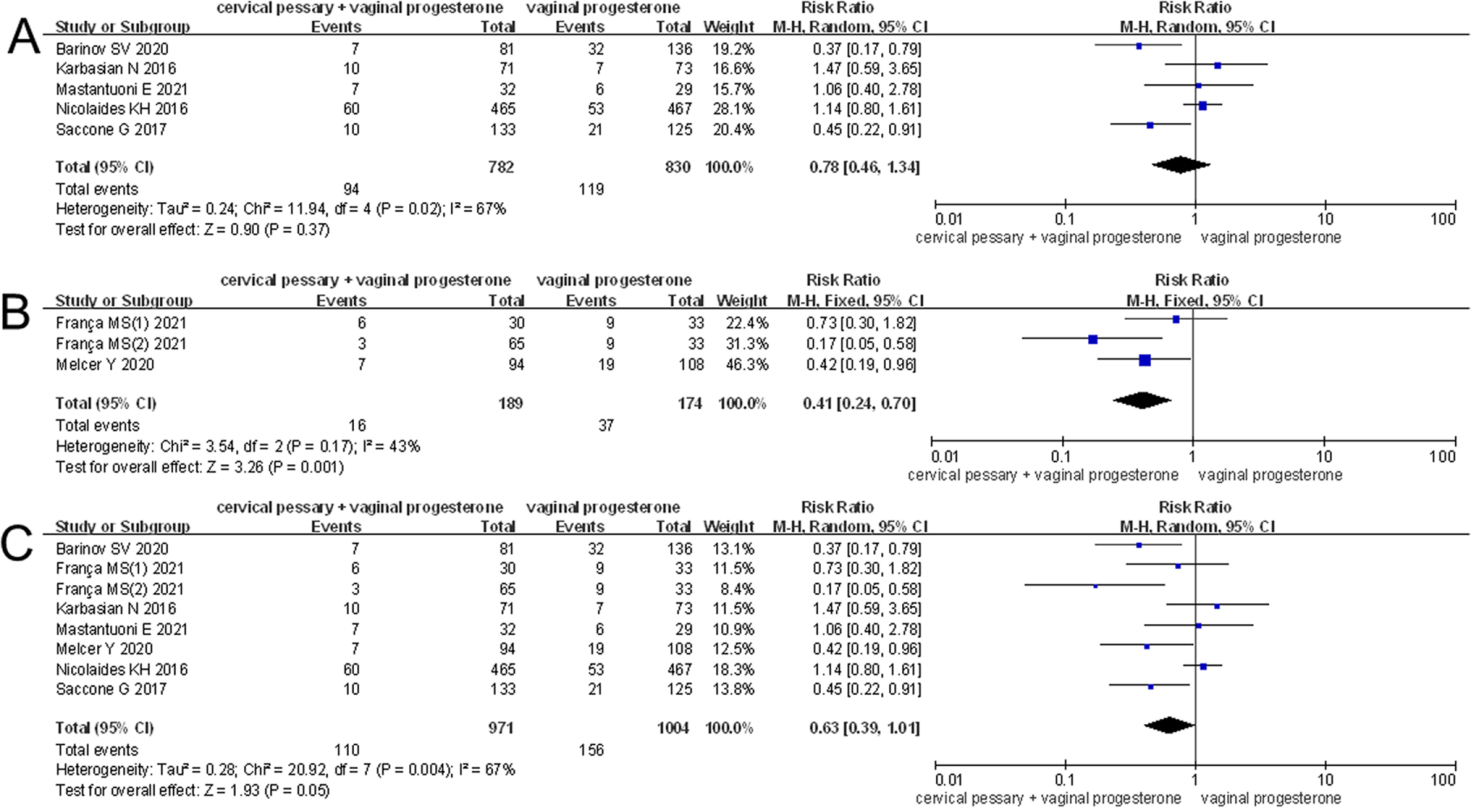
Forest plot to assess the efficacy of cervical pessary plus vaginal progesterone compared to the vaginal progesterone alone treatment group. **A**–**C** Meta-analysis of RCTs (**A**, a random-effects model), non-RCT (**B**, a fixed-effects model), and total trials (**C**, a random-effects model) to observe the effects on preventing preterm birth before 34 weeks of gestation in singleton pregnancies. RCTs, randomized controlled trials; CI, confidence interval

Meta-analysis of five RCTs showed that compared with vaginal progesterone alone, cervical pessary + vaginal progesterone combined treatment did not further reduce the risk of PTB < 28 weeks (RR = 1.54, 95% CI: 0.87–2.74, *p* = 0.14), < 32 weeks (RR = 1.32, 95% CI: 0.89–1.97, *p* = 0.16), < 34 weeks (RR = 0.78, 95% CI: 0.46–1.34, *p* = 0.37; Fig. 2A), and < 37 weeks (RR = 1.09, 95% CI: 0.52–2.27, *p* = 0.82) of gestation (Table 3). There were also no differences in mean GA at delivery, incidence of LBW, birth weight, composite adverse neonatal outcomes (including antenatal death, neonatal death, perinatal death, IVH, RDS, ROP, sepsis), and NICU admission between two groups (*p* > 0.05; Table 3).

**Table 3:** Effects of cervical pessary plus vaginal progesterone use on pregnancy and neonatal outcomes in singleton gestations

Comparison	Design	Outcomes	No	ES (95%CI)	P_E_-value	I^2^	P_H_-value	Model
Cervical pessary + vaginal progesterone vs vaginal progesterone	RCT	PTB < 28 weeks of gestation	2	1.54 (0.87,2.74)	0.14	0.0	0.46	Fixed-effects
		PTB < 32 weeks of gestation	2	1.32 (0.89,1.97)	0.16	0.0	0.96	Fixed-effects
		PTB < 34 weeks of gestation	5	0.78 (0.46,1.34)	0.37	66.5	0.02	Random-effects
		PTB < 37 weeks of gestation	3	1.09 (0.52,2.27)	0.82	74.1	0.02	Random-effects
		GA at delivery	3	0.11 (− 0.16,0.37)	0.44	58.7	0.09	Random-effects
		Incidence of LBW	2	1.09 (0.85,1.39)	0.50	0.0	0.49	Fixed-effects
		Birth weight	2	− 0.20 (− 0.48,0.07)	0.15	0.0	0.85	Fixed-effects
		Antenatal death	2	1.34 (0.47,3.85)	0.58	0.0	0.84	Fixed-effects
		Neonatal death	2	1.32 (0.48,3.64)	0.59	0.0	0.37	Fixed-effects
		Perinatal death	2	1.32 (0.65,2.69)	0.45	0.0	0.79	Fixed-effects
		NICU admission	2	1.23 (0.81,1.87)	0.32	0.0	0.63	Fixed-effects
		IVH	2	2.53 (0.80,7.94)	0.11	0.0	0.42	Fixed-effects
		RDS	2	1.21 (0.74,1.99)	0.46	0.0	0.61	Fixed-effects
		ROP	2	4.32 (0.74,25.13)	0.10	0.0	0.74	Fixed-effects
		Sepsis	2	1.40 (0.82,2.38)	0.22	0.0	1.00	Fixed-effects
		Composite adverse neonatal outcomes	4	1.15 (0.79,1.70)	0.47	0.0	0.85	Fixed-effects
	Non-RCT	**PTB < 34 weeks of gestation**	**3**	**0.41 (0.24,0.70)**	**0.001**	**43.5**	**0.17**	**Fixed-effects**
		PTB < 37 weeks of gestation	2	1.06 (0.76,1.48)	0.72	0.0	0.41	Fixed-effects
		Birth weight	2	0.02 (− 0.17,0.21)	0.81	0.0	0.32	Fixed-effects
		Vaginal discharge	2	3.13 (0.06,173.93)	0.58	96.8	< 0.001	Random-effects
	Total	PTB < 28 weeks of gestation	3	1.17 (0.52,2.64)	0.70	25.2	0.26	Fixed-effects
		PTB < 34 weeks of gestation	8	0.63 (0.39,1.01)	0.05	66.5	0.004	Random-effects
		PTB < 37 weeks of gestation	5	1.07 (0.72,1.58)	0.74	54.8	0.07	Random-effects
		GA at delivery	4	0.09 (− 0.12,0.29)	0.42	57.4	0.07	Random-effects
		Birth weight	4	− 0.05 (− 0.20,0.11)	0.54	0.0	0.43	Fixed-effects
Cervical pessary + vaginal progesterone vs cervical cerclage + vaginal progesterone	Non-RCT	PTB < 37 weeks of gestation	2	0.73 (0.53,1.01)	0.06	0.0	0.34	Fixed-effects
		Birth weight	2	− 0.02 (− 0.24,0.20)	0.87	0.0	0.41	Fixed-effects
Cervical pessary + vaginal progesterone vs vaginal progesterone, cervical cerclage and pessary	Non-RCT	PTB < 37 weeks of gestation	1	0.73 (0.41,1.30)	0.29	-	-	Fixed-effects
		GA at delivery	1	0.24 (− 0.26,0.74)	0.35	-	-	Fixed-effects
		**Birth weight**	**1**	**0.53 (0.03,1.03)**	**0.04**	**-**	**-**	**Fixed-effects**
Cervical pessary + vaginal progesterone vs tocolysis	Non-RCT	PTB < 32 weeks of gestation	1	0.14 (0.01,2.47)	0.18	-	-	Fixed-effects
		PTB < 34 weeks of gestation	1	0.07 (0.004,1.08)	0.06	-	-	Fixed-effects
		**PTB < 37 weeks of gestation**	**1**	**0.23 (0.07,0.74)**	**0.01**	**-**	**-**	**Fixed-effects**
		**GA at delivery**	**1**	**0.61 (0.20,1.03)**	**0.004**	**-**	**-**	**Fixed-effects**
		**Birth weight**	**1**	**0.64 (0.23,1.05)**	**0.002**	**-**	**-**	**Fixed-effects**
		**Composite adverse neonatal outcomes**	**1**	**0.11 (0.03,0.45)**	**0.002**	**-**	**-**	**Fixed-effects**
		RDS	1	0.07 (0.004,1.08)	0.06	-	-	Fixed-effects
		**Vaginal discharge**	**1**	**89.59 (5.66,1418.63)**	**0.001**	**-**	**-**	**Fixed-effects**
Cervical pessary + vaginal progesterone vs pessary	Non-RCT	PTB < 28 weeks of gestation	1	1.67 (0.42,6.62)	0.47	-	-	Fixed-effects
		PTB < 32 weeks of gestation	1	1.38 (0.60,3.15)	0.45	-	-	Fixed-effects
		PTB < 34 weeks of gestation	1	1.31 (0.71,2.42)	0.39	-	-	Fixed-effects
		PTB < 37 weeks of gestation	1	1.04 (0.68,1.60)	0.85	-	-	Fixed-effects
		Composite adverse neonatal outcomes	1	1.25 (0.54,2.92)	0.61	-	-	Fixed-effects
		NICU admission	1	1.00 (0.55,1.83)	1.00	-	-	Fixed-effects
		Birth weight	1	− 0.03 (− 0.41,0.35)	0.88	-	-	Fixed-effects

Statistically significant results for treatment are given in bold

*RCT* randomized controlled trials, *PTB* preterm birth, *GA* gestational age, *LBW* low birth weight, *NICU* neonatal intensive care unit, *IVH* intraventricular hemorrhage, *RDS* respiratory distress syndrome, *ROP* retinopathy of prematurity, *ES* effect size, *CI* confidence interval, *P*_*H*_*-value* significance for heterogeneity, *P*_*E*_*-value* significance for treatment effects

**Fig. 3:**
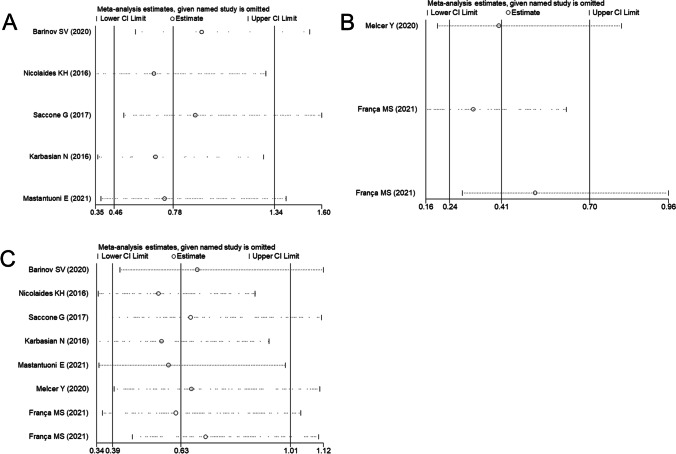
Sensitivity analysis for RCTs (**A**), non-RCT (**B**), and total trials (**C**) to observe the effects on preventing preterm birth < 34 weeks of gestation in singleton pregnancies. RCTs, randomized controlled trials; CI, confidence interval

**Fig. 4:**
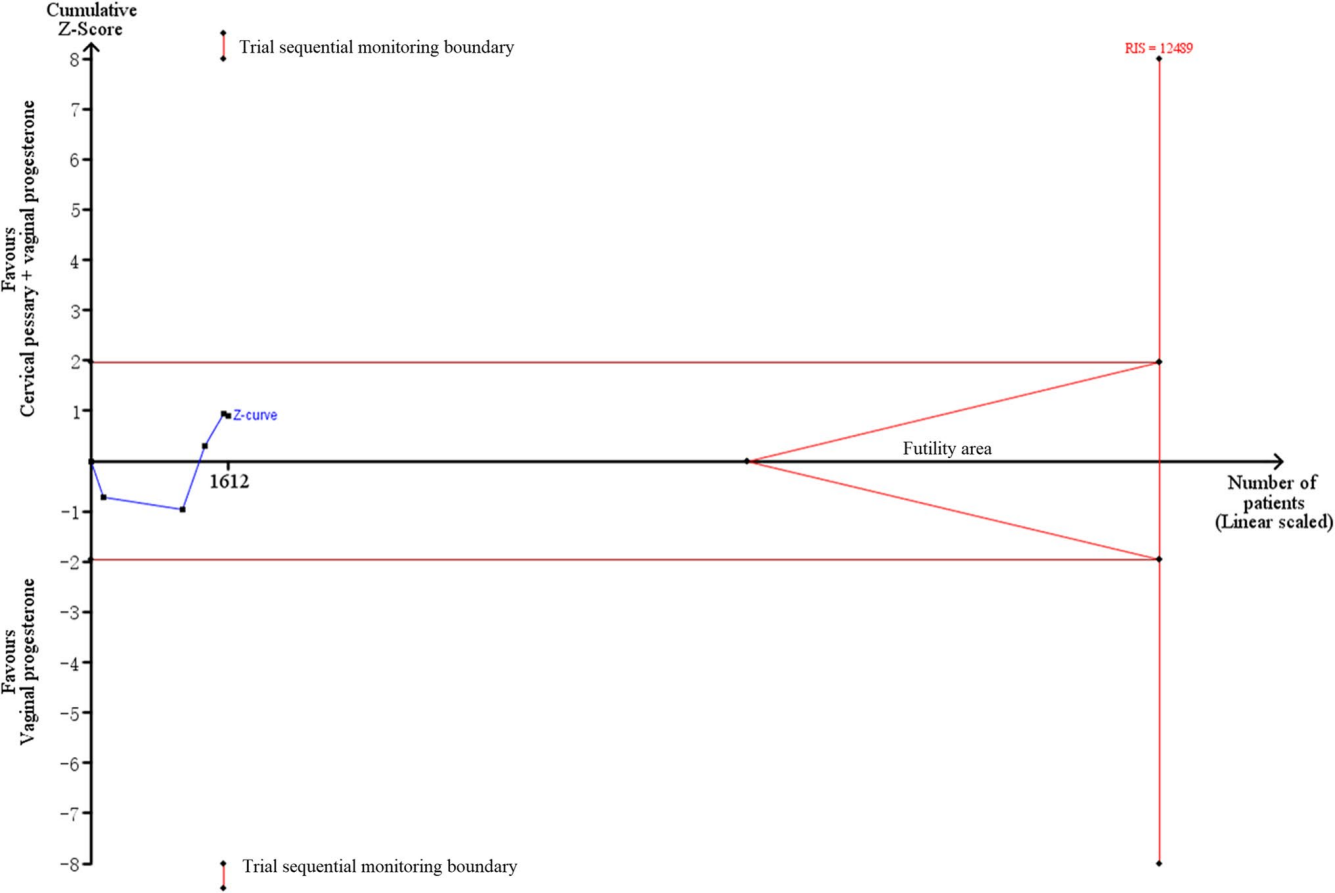
Trial sequential analysis for the effects of cervical pessary plus vaginal progesterone on preterm birth < 34 weeks of gestation in singleton pregnancies using five RCTs. RCTs, randomized controlled trials; RIS, required information size

Subgroup analysis nearly did not change the results for PTB < 34 weeks of gestation and composite adverse neonatal outcomes except cervical length > 25 mm (RR = 0.37, 95% CI: 0.17–0.79, *p* = 0.01) (Table 4). Egger’s linear regression test showed no evidence of publication bias for PTB < 34 weeks of gestation (*p* = 0.48), and sensitivity analysis showed the removal of any one study did not affect the pooled results (Fig. 3A). However, TSA results indicated a possibility of false positivity because the cumulative Z-curve did not enter the futility area, reach RIS or cross TSMB (TSA-adjusted CI 0.09 to 7.00; Fig. 4). Thus, additional studies are still required to reach a firm conclusion.

**Table 4:** Subgroup analysis for singleton gestations

Subgroup			No	ES (95%CI)	P_E_-value	I^2^	P_H_-value	Model
PTB < 34 weeks of gestation (RCT)	Cervical length	≤ 25 mm	4	0.94 (0.57,1.53)	0.80	51.1	0.11	Random-effects
		** > 25 mm**	**1**	**0.37 (0.17,0.79)**	**0.01**	**-**	**-**	**Random-effects**
	Ethnicity	Asian	1	1.47 (0.59,3.65)	0.41	-	-	Random-effects
		Non-Asian	4	0.68 (0.37,1.28)	0.23	71.9	0.01	Random-effects
	Design	Multiple-center	1	1.14 (0.80,1.61)	0.47	-	-	Random-effects
		Single-center	4	0.68 (0.35,1.29)	0.23	58.6	0.06	Random-effects
Composite adverse neonatal outcomes (RCT)	Cervical length	≤ 25 mm	4	1.15 (0.79,1.70)	0.47	0.0	0.85	Fixed-effects
		> 25 mm	0	-	-	-	-	-
	Ethnicity	Asian	1	1.03 (0.15,7.10)	0.98	-	-	Fixed-effects
		Non-Asian	3	1.16 (0.78,1.72)	0.46	0.0	0.67	Fixed-effects
	Design	Multiple-center	2	1.23 (0.80,1.88)	0.34	0.0	0.74	Fixed-effects
		Single-center	2	0.82 (0.32,2.10)	0.68	0.0	0.78	Fixed-effects
PTB < 34 weeks of gestation (total)	Cervical length	≤ 25 mm	7	0.69 (0.42,1.12)	0.79	64.4	0.01	Random-effects
		** > 25 mm**	**1**	**0.37 (0.17,0.79)**	**0.01**	**-**	**-**	**Random-effects**
	Ethnicity	Asian	1	1.47 (0.59,3.65)	0.41	-	-	Random-effects
		**Non-Asian**	**7**	**0.56 (0.34,0.93)**	**0.03**	**68.2**	**0.004**	**Random-effects**
	Design	Multiple-center	2	0.75 (0.29,1.95)	0.55	78.9	0.03	Random-effects
		Single-center	6	0.58 (0.33,1.01)	0.05	56.5	0.04	Random-effects

Statistically significant results for treatment are given in bold (analysis with two or more studies)

*RCT* randomized controlled trials, *PTB* preterm birth, *ES* effect size, *CI* confidence interval, *P*_*H*_*-value* significance for heterogeneity, *P*_*E*_*-value* significance for treatment effects

#### Cervical Pessary + Vaginal Progesterone vs Vaginal Progesterone: Non-RCTs

**Fig. 5:**
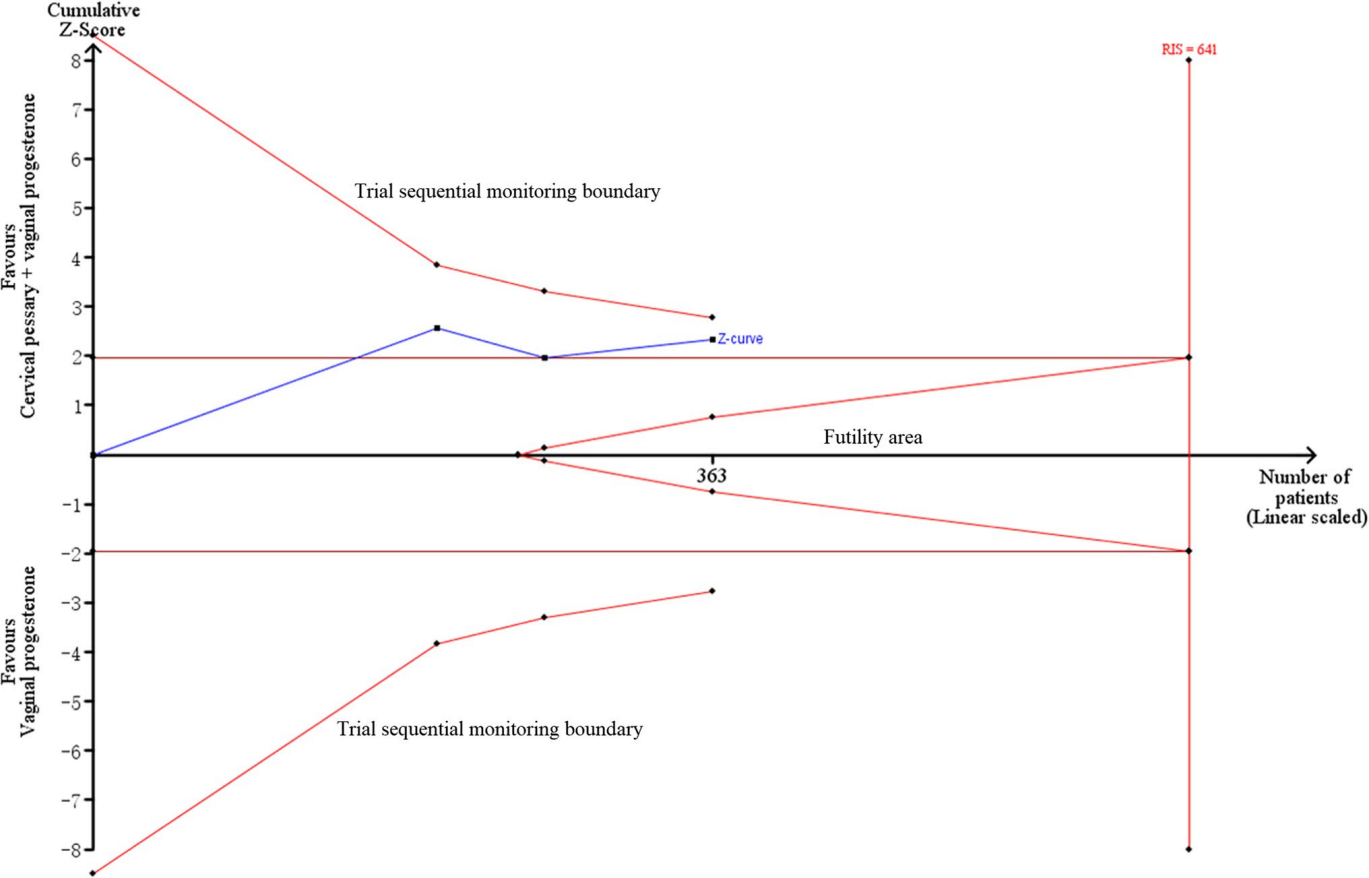
Trial sequential analysis for the effects of cervical pessary plus vaginal progesterone on preterm birth < 34 weeks of gestation in singleton pregnancies using three non-RCTs. RCTs, randomized controlled trials; RIS, required information size

Meta-analysis of three non-RCTs revealed that cervical pessary + vaginal progesterone combined treatment had significant effectiveness on preventing PTB < 34 weeks of gestation (RR = 0.41, 95% CI: 0.24–0.70, *p* = 0.001) (Fig. 2B). Heterogeneity (*I*^2^ = 43.5%, *p* = 0.17; Table 3) and publication bias (*p* = 0.47) were both absent, suggesting robust effects of cervical pessary + vaginal progesterone combined treatment, which was also confirmed in sensitivity analysis (Fig. 3B). However, the cumulative *Z*-curve did not enter the futility area, reach the RIS or cross TSMB (TSA-adjusted CI 0.14 to 1.18; Fig. 5), indicating the meta-analysis results remained inconclusive.

#### Cervical Pessary + Vaginal Progesterone vs Vaginal Progesterone: RCT and Non-RCTs

**Fig. 6:**
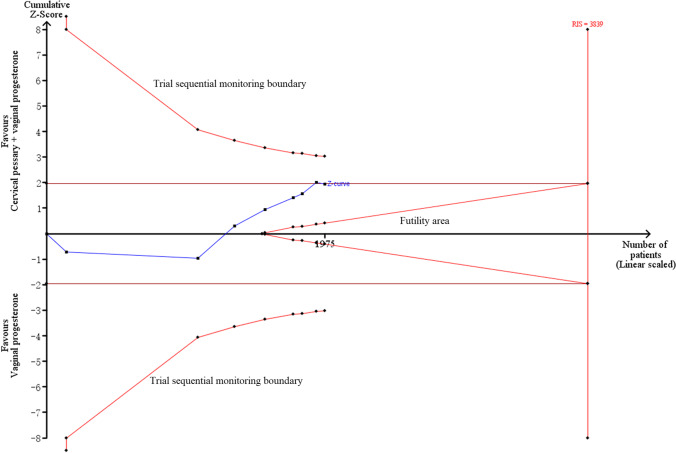
Trial sequential analysis for the effects of cervical pessary plus vaginal progesterone on preterm birth < 34 weeks of gestation in singleton pregnancies using five RCTs and three non-RCTs. RCTs, randomized controlled trials; RIS, required information size

Meta-analysis of total RCTs and non-RCTs indicated a marginal significance was achieved in reducing the risk of PTB < 34 weeks of gestation (RR = 0.63, 95% CI: 0.39–1.01, *p* = 0.05) by cervical pessary + vaginal progesterone combined treatment when comparing with vaginal progesterone alone (Fig. 2C). This significance was further improved in non-Asian (RR = 0.56, 95% CI: 0.34–0.93, *p* = 0.03) and a normal cervical length (> 25 mm; RR = 0.37, 95% CI: 0.17–0.79, *p* = 0.01) subgroups (Table 4). There was no evidence of publication bias (*p* = 0.11), and sensitivity analysis demonstrated that the pooled results were robust (Fig. 3C). The TSA showed that there was not enough information to confirm this conclusion (TSA-adjusted CI 0.31 to 1.30; Fig. 6).

#### Cervical Pessary + Vaginal Progesterone vs Cervical Cerclage + Vaginal Progesterone

Meta-analysis of two trials by a fixed-effect model revealed that there were no differences in PTB < 37 weeks of gestation (RR = 0.73, 95% CI: 0.53–1.01, *p* = 0.06) and birth weight (SMD =  − 0.02, 95% CI: − 0.24–0.20, *p* = 0.87) when comparing cervical pessary + vaginal progesterone with cervical cerclage + vaginal progesterone (Table 3). Also, cerclage was an invasive surgery, while cervical pessary is a flexible, ring-like, silicon device that is directly inserted and removed in the vagina of pregnant women without anesthesia and thus is relatively non-invasive. Thus, we consider cervical pessary + vaginal progesterone combined treatment may be more effective and safe.

#### Cervical Pessary + Vaginal Progesterone vs Vaginal Progesterone + Cervical Cerclage + Pessary

Only one study compared the effects of cervical pessary + vaginal progesterone with a three-combined method (vaginal progesterone + cervical cerclage + pessary) [18]. The calculated RR and 95% CI showed cervical pessary + vaginal progesterone achieved the same effects with the three-combined method, without significance in preventing PTB < 37 weeks of gestation (*p* = 0.29) or shortening GA at delivery (*p* = 0.35) (Table 3). Even, birth weight of neonate was higher (SMD = 0.53, 95% CI: 0.03–1.03, *p* = 0.04) (Table 3). These findings indicated cervical pessary + vaginal progesterone may be enough to prevent PTB, while cervical cerclage may be unnecessary.

#### Cervical Pessary + Vaginal Progesterone vs Tocolysis

Tajima et al. compared the effects of cervical pessary + vaginal progesterone with ritodrine hydrochloride and magnesium sulfate-based tocolysis [22]. The results showed cervical pessary + vaginal progesterone may be more effective in preventing PTB < 37 weeks of gestation (RR = 0.23, 95% CI: 0.07–0.74, *p* = 0.01) and composite adverse neonatal outcomes (RR = 0.11, 95% CI: 0.03–0.45, *p* = 0.002), increasing GA at delivery (SMD = 0.61, 95% CI: 0.20–1.03, *p* = 0.004) and birth weight (SMD = 0.64, 95% CI: 0.23–1.05, *p* = 0.002) (Table 3).

#### Cervical Pessary + Vaginal Progesterone vs Pessary

Stricker et al. compared the effects of cervical pessary + vaginal progesterone with pessary alone [8]. Unexpected, no significant difference was observed in PTB < 28 weeks, < 32 weeks, PTB < 34 weeks, PTB < 37 weeks of gestation, composite adverse neonatal outcomes, NICU admission, and birth weight (*p* > 0.05; Table 3). However, due to the fact that the mean duration of stay in the NICU was shorter in the combined group than that in the pessary group (46.5 days vs 52.0, *p* < 0.01) [8], we considered cervical pessary + vaginal progesterone may be more beneficial.

### Analysis for Outcomes in Women with Multiple Pregnancies

#### Cervical Pessary + Vaginal Progesterone vs Vaginal Progesterone: RCTs + Non-RCTs

Meta-analysis of two non-RCTs showed compared with vaginal progesterone, cervical pessary + vaginal progesterone combined treatment had no significant effectiveness to influence PTB < 34 weeks of gestation, but significantly increased neonatal birth weight (SMD = 0.50, 95% CI: 0.15–0.85, *p* = 0.01) under a fixed-effect model (*I*^2^ = 0%, *p* = 0.67; Table 5).

**Table 5:** Effects of cervical pessary plus vaginal progesterone use on pregnancy and neonatal outcomes in multiple gestations

Comparison	Design	Outcomes	No	ES (95%CI)	P_E_-value	I^2^	P_H_-value	Model
Cervical pessary + vaginal progesterone vs vaginal progesterone	Total	PTB < 34 weeks of gestation	2	0.83 (0.57,1.22)	0.34	43.0	0.35	Fixed-effects
		GA at delivery	1	0.47 (− 0.03,0.97)	0.07	-	-	Fixed-effects
		**Birth weight**	**2**	**0.50 (0.15,0.85)**	**0.01**	**0.0**	**0.67**	**Fixed-effects**
		Perinatal death	1	0.32 (0.02,5.77)	0.44	-	-	Fixed-effects
		Composite adverse neonatal outcomes	1	0.27 (0.07,1.06)	0.06	-	-	Fixed-effects
Cervical pessary + vaginal progesterone vs conservative	Non-RCT	GA at delivery	1	0.28 (0.03,2.58)	0.26	-	-	Fixed-effects
		PTB < 28 weeks of gestation	**1**	**0.27 (0.08,0.90)**	**0.03**	**-**	**-**	**Fixed-effects**
Cervical pessary + vaginal progesterone vs no treatment	Non-RCT	PTB < 28 weeks of gestation	1	0.25 (− 0.27,0.77)	0.34	-	-	Fixed-effects
		PTB < 32 weeks of gestation	1	1.71 (0.42,6.94)	0.46	-	-	Fixed-effects
		PTB < 34 weeks of gestation	1	2.13 (0.90,5.07)	0.09	-	-	Fixed-effects
		GA at delivery	1	− 0.18 (− 0.71,0.34)	0.49	-	-	Fixed-effects

Statistically significant results for treatment are given in bold

*RCT* randomized controlled trials, *PTB* preterm birth, *GA* gestational age, *NICU* neonatal intensive care unit, *LBW* low birth weight, *ES* effect size, *CI* confidence interval, *P*_*H*_*-value* significance for heterogeneity, *P*_*E*_*-value* significance for treatment effects

#### Cervical Pessary + Vaginal Progesterone vs Conservative

Zimerman et al. compared the effects of cervical pessary + vaginal progesterone with conservative treatment [24]. The results showed the incidence of PTB < 28 weeks of gestation was significantly reduced (RR = 0.27, 95% CI: 0.08–0.90, *p* = 0.03; Table 5), implying the importance to use cervical pessary + vaginal progesterone.

#### Cervical Pessary + Vaginal Progesterone vs No Treatment

In the study of França et al. [17], GA at delivery, PTB < 28 weeks, PTB < 32 weeks, and PTB < 34 weeks of gestation in cervical pessary plus progesterone group was suggested to be equivalent to the non-treated group (*p* > 0.05; Table 5).

## Discussion

There were three systematic review and meta-analysis studies exploring the effectiveness of a combined use of cervical pessary and vaginal progesterone [13, 14, 31] previously, but the number of their enrolled articles was small: Jarde et al. integrated one RCT (singleton, vs vaginal progesterone) [10] and two cohort studies (singleton, vs pessary [8], twin, vs vaginal progesterone [9]); Liu et al. [13] and Conde-Agudelo et al. [31] included three RCTs (all singleton, vs vaginal progesterone) [10–12], which consequentially led to the limited outcomes to be analyzed (results of PTB at < 34 weeks of gestation available in three studies [13, 14, 31], LBW delivery, perinatal death and NICU admission only in the study of Liu et al. [13]). Compared with these studies, our study further increased the statistical power by including ten studies with patients in singleton gestations (five RCTs, vs vaginal progesterone; four cohorts, vs vaginal progesterone; two cohorts, vs cervical cerclage + vaginal progesterone) and two cohort studies with patients in multiple gestations (vs vaginal progesterone) for meta-analysis, which also led to more outcomes analyzable. The other four studies vs other controls (such as pessary, three combined, tocolysis, conservative or no treatment; only one study for each) were not integrated, but independently analyzed to prevent the heterogeneity. Thus, our conclusion may be more believable.

Although similar to previous studies [13, 14, 31], our meta-analysis of RCTs showed compared with vaginal progesterone alone, cervical pessary plus vaginal progesterone did not reduce the risk of PTB < 34 weeks of gestation, meta-analysis of retrospective cohort studies and total trials (especially non-Asian and normal cervical length subgroups) revealed this combined treatment was significantly associated with a lower risk of PTB < 34 weeks of gestation in singleton women. Also, the effect size (non-RCT, 0.41; non-Asian, 0.56; cervical length > 25 mm, 0.37) of this combined therapy was larger than that in some meta-analyses or individual studies exploring the progesterone as a single agent regardless of cervical length (Romero et al.: RR = 0.62 [32]; EPPPIC group: RR = 0.78 [33]; Hassan et al.: RR = 0.51 [34]; Phung et al.: RR = 0.51 [35]; Norman et al.: RR = 0.86 [36, 37]; the later three were not significant at *p* value). Furthermore, cervical pessary plus vaginal progesterone had no difference in preventing PTB compared with pessary, invasive cervical cerclage + vaginal progesterone or invasive cervical cerclage + vaginal progesterone + pessary, but had superior effects than tocolysis (Table 3). Although cervical pessary plus vaginal progesterone seemed to increase a higher risk of vaginal discharge (compared with tocolysis), this discharge may be mainly attributed to the response of vaginal glands to a foreign body (pessary) and may be not harmful to the pregnancy. These findings reveal cervical pessary plus vaginal progesterone may be a non-invasive, inexpensive, effective and safe treatment approach for reducing the risk of PTB in singleton pregnancies.

Our study was the first to use the systematic review and meta-analysis to explore the effects of cervical pessary plus vaginal progesterone for multiple pregnancies. Our results showed cervical pessary plus vaginal progesterone did not reduce the risk of PTB < 34 weeks of gestation, but enhanced the neonatal birth weight at significant levels (SMD = 0.50, *p* = 0.01), increased GA at delivery (RR = 0.47, *p* = 0.07) and decreased the incidence of composite adverse neonatal outcomes at marginal significance levels (RR = 0.27, *p* = 0.06) compared with vaginal progesterone alone. The negative effect of our combined treatment for PTB < 34 weeks was similar to vaginal progesterone alone treatment (compared with placebo) as reported in a previous meta-analysis [38]. The effects of our combined treatment on neonatal birth weight, GA at delivery and composite adverse neonatal outcomes were obviously superior to vaginal progesterone alone treatment (which was shown to have no differences relative to placebo [39, 40], even if doses were increased [41]). Furthermore, cervical pessary plus vaginal progesterone was also observed to reduce the risk of PTB < 28 weeks of gestation compared with conservative treatment, but have no difference with cervical cerclage + vaginal progesterone (Table 5). These findings reveal cervical pessary plus vaginal progesterone may be potentially effective and safe for preventing preterm delivery in multiple pregnancies.

Progesterone was reported to prevent cervical dilation, PTB, and reduce the mortality of neonates by downregulating the expression of several pro-inflammatory mediators induced by activation of T-cells in the maternal–fetal interface (CASP11, CCL-22, ICAM1, CTLA4, NOD1, and CCL5), myometrium (IL-33), and cervical tissues (IL-33, IL-6, IL-12b, IL-1a, PYCARD, IL-4) [42]. Progesterone acts the anti-inflammatory roles and anti-cervical ripening by activation of progesterone receptor [43]. Although the exact mechanisms to explain the contribution of the cervical pessary to prolong pregnancy remain unclear, it may be associated with the following reasons: (1) mechanically changing the utero-cervical angle (defined as the distance between the anterior uterine wall and the axis of the cervical canal) to make it more acute [19, 44, 45] and then preventing direct pressure on the internal cervical os and cervix itself [46]; (2) preventing further opening of the internal os due to dissociation of amnion and chorion, particularly when the pregnant woman was in the upright position [47]; (3) protecting the cervical mucus plug through supporting the attachment of the remaining cervical tissue [46]; and (4) supporting the immunological barrier between the chorioamnion-extraovular space and the vaginal microbiological flora [48].

Our study has some limitations. First, the number of included studies is still limited and the sample size is still relatively small (especially for multiple pregnancies). According to the TSA results, the RIS was not reached for all our therapeutic outcomes in meta-analysis. Only one study was included to explore the therapeutic difference of cervical pessary plus vaginal progesterone compared with pessary alone [8], tocolysis [22], conservative [24], or no treatment [17], leading to meta-analysis inexecutable and only a preliminary systematic review for them. If the sample size was larger, the conclusions of these systematic review articles may be different and should be cautiously interpreted. Second, some studies were retrospective and non-randomized that may cause selection bias and influence the results. Third, the heterogeneity was present within the included trials, especially the RCTs. Saccone et al. [11] only recruited women without prior PTB (in which combined therapy was shown to be effective), while the other studies [10, 12, 25] included women with and without prior PTB (all showed non-difference between combined and single treatment). Cervical length was normal in the study of Barinov et al. [19] (in which therapeutic result was significant), while the women in other studies had short cervix [10, 11, 25] (non-significant). França et al. [26] also demonstrated that the learning process was present using the cervical pessary. Combined cervical pessary performed by a trained and experienced physician can achieve significant results, but not for inexperienced doctors. These may be the underlying reason to explain the negative results in meta-analysis of RCTs. Therefore, more RCTs with consistent inclusion criteria should be designed to further confirm the therapeutic effects of cervical pessary plus vaginal progesterone and stratify women who could benefit from additional pessaries. For some pregnancies, it is likely that vaginal progesterone alone was already sufficient in risk mitigation, so any additional or marginal benefit of other interventions would be difficult to discern [12]. Fourth, progesterone can be natural or synthetic (17alpha-hydroxyprogesterone caproate) and administered orally, intramuscularly, vaginally, or rectally [49]; some meta-analysis showed that there were differences in the efficacy of different routes or source [50, 51]. However, only vaginal natural progesterone was reported to be combined with pessary and included in our study. Thus, in the future, trials that investigate the effects of cervical pessary plus synthetic progesterone via various routes should also be scheduled to identify the most suitable combined therapy strategy. Fifth, we consider cervical pessary plus progesterone may be an effective and safe combined therapy approach, and mainly address its superiority by comparing with other treatments. However, it is still necessary to use a network meta-analysis [51] to study all combined methods to ultimately achieve a robust conclusion when the evidence is sufficient.

## Conclusion

This comprehensive synthesis of the literature suggests that a combined use of cervical pessary and vaginal progesterone may be safe and effective to prevent PTB before 34 weeks in singleton pregnancies and increase the neonatal birth weight in the multiple pregnancies compared with vaginal progesterone alone. More RCTs studies with consistent design should be performed to confirm the efficacy of this therapeutic strategy.

## Supplementary Information

Below is the link to the electronic supplementary material.

Supplementary file1 (DOCX 21 KB)

Supplementary file2. Table S2 Analyzed data for each outcome. (XLSX 31 KB)

## Data Availability

All data generated or analyzed during this study are included in this published article.
