# Supplementary material for: Prevention of Preterm Birth by Cervical Pessary Combined with Vaginal Progesterone: a Systematic Review and Meta-analysis with Trial Sequential Analysis
Source: Reprod Sci. 2022 Mar 29;30(1):93–110. doi: 10.1007/s43032-022-00926-x (PMC9810688; doi:10.1007/s43032-022-00926-x)
Supplement: Supplementary file 1 — Supplementary file1 (DOCX 21 KB) [file 43032_2022_926_MOESM1_ESM.docx]

Table S1 Definition of potential risk for preterm birth

|  | Study | Year | Inclusion criteria of patients |
| --- | --- | --- | --- |
|  |  |  |  |
| Meta | Mastantuoni E[25] | 2021 | Arrested preterm labor a cervical length of ≤ 25 mm |
|  | Barinov SV[19] | 2020 | A history of spontaneous miscarriage at 14−21 weeks and/or preterm birth at 22−37 weeks of gestation, or pregnancy after in vitro fertilization |
|  | Saccone G[11] | 2017 | A cervical length ≤ 25 mm at 18 weeks 0 days to 23 weeks 6 days of gestation |
|  | Karbasian N[12] | 2016 | A cervical length ≤ 25 mm at 18–22 gestational weeks |
|  | Nicolaides KH [10] | 2016 | A cervical length of ≤ 25 mm at 20 weeks 0 days to 24 weeks 6 days of gestation |
|  | França MS [26] | 2021 | A cervical length ≤ 25 mm between 18th and 24th week |
|  | Firichenko SV [27] | 2021 | A cervical length ≤ 25 mm |
|  | Shor S[18] | 2019 | A cervical length ≤ 25 mm performed between 15-29 weeks gestation and delivered >24 weeks of gestation |
|  | Melcer Y[21] | 2020 | A cervical length ≤ 25 mm performed between 15-29 weeks gestation and delivered >24 weeks of gestation |
|  | Shor S[18] | 2019 | A cervical length ≤ 25 mm performed between 15-29 weeks gestation and delivered >24 weeks of gestation |
|  | Barinov SV[20] | 2021 | History of spontaneous miscarriage between 14−21 weeks of gestation; history of preterm labor between 22−37 weeks of gestation; pregnancy after in vitro fertilization; large uterine fibroids, including fibroids of atypical location; placental praevia; confirmed cervical incompetence (length of the closed cervical part ≤25 mm and/or dilation of the cervical canal to ≥ 10 mm) |
|  | Fox NS [9] | 2016 | Twin pregnancies with a cervical length ≤ 20 mm |
|  | Yaniv-Nachmani H [23] | 2021 | Twin gestations and short cervical length (< 25 mm) between 16-28 weeks' gestation |
| Systematic review | Shor S[18] | 2019 | A cervical length ≤ 25 mm performed between 15-29 weeks gestation and delivered >24 weeks of gestation |
|  | Tajima M[22] | 2020 | A cervical length ≤ 25 mm at 20–24 weeks or ≤ 20 mm at 25–34 weeks |
|  | Stricker N [8] | 2015 | A history of spontaneous preterm birth, surgical conization, cervical length < 3rd centile or percentile |
|  | Barinov SV[31] | 2017 | High risk of miscarriage and preterm labor (history of miscarriage or preterm labor); confirmed cervical insufficiency before 24 weeks of pregnancy (cervical length < 25mm); history of reconstructive cervical surgery (after conization or cervical scars); moderate to large uterine fibroids; pregnancy after in vitro fertilization; and multiple pregnancy |
|  | Zimerman A [24] | 2018 | Twin pregnancies with a cervical length ≤ 25mm in second trimester 16-28 gestational week |
|  | França MS [17] | 2020 | A cervical length < 25 mm during gestational age between 18 to 27 weeks and 6 days |
